# Manganese Oxide as an Electrochemical Sensor for Carbamazepine and Sulfamethoxazole in Wastewater Samples

**DOI:** 10.1002/open.202500520

**Published:** 2026-01-22

**Authors:** Pheladi Lizzy Mokaba, Collen Nepfumbada, Boipelo Nichollette Mathe, Aitor Larranaga, Ntuthuko Wonderboy Hlongwa, Usisipho Feleni

**Affiliations:** ^1^ Institute for Nanotechnology and Water Sustainability (iNanoWS) College of Science, Engineering and Technology Roodepoort University of South Africa South Africa; ^2^ Group of Science and Engineering of Polymeric Biomaterials (ZIBIO Group) Department of Mining, Metallurgy Engineering and Materials Science Bilbao School of Engineering POLYMAT University of the Basque Country (UPV/EHU) Bilbao Spain

**Keywords:** carbamazepine, electrochemical sensor, environmental monitoring, MnO_2_ nanoparticles, sulfamethoxazole, wastewater analysis

## Abstract

The extensive use of pharmaceutical compounds poses a growing threat to environmental and public health. Carbamazepine (CBZ) and sulfamethoxazole (SMX), widely used in veterinary and human medicine, are persistent pollutants often detected in water bodies. Their presence at trace levels can contribute to the development of antibiotic resistance. In this study, a novel electrochemical sensor based on manganese oxide nanoparticles (MnO_2_NPs) modified screen‐printed carbon electrode (SPCE) was fabricated for the detection of CBZ and SMX. The effects of pH, scan rate, and analyte concentration were systematically investigated. Under optimized conditions, the sensor exhibited excellent sensitivity with detection limits of 0.106 nanomolar (CBZ) and 0.082 nanomolar (SMX), respectively within a linear range of 0.97–5.82 nanomolar. The sensor showed outstanding selectivity and stability, and its effectiveness was confirmed by recovery tests in real wastewater samples, with values ranging from 95% to 110% (CBZ) and 90% to 105% (SMX), respectively. These findings demonstrate the practical potential of MnO_2_NPs/SPCE‐based sensors for monitoring emerging contaminants.

## Graphical abstract

A manganese oxide (MnO_2_) nanoparticle‐modified screen‐printed carbon electrode enables sensitive and simultaneous electrochemical detection of carbamazepine and sulfamethoxazole in wastewater, demonstrating improved oxidation signals and enhanced analytical performance for pharmaceutical monitoring.

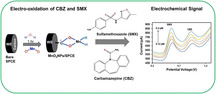

## 1 Introduction

Pharmaceuticals have increasingly become recognized as contaminants of emerging concern, as their unintended release into the environment poses significant risks to both aquatic ecosystems and human health [1]. These compounds often escape regulation and their long‐term ecotoxicological effects remain poorly understood [2]. Among the most ubiquitous pharmaceuticals detected in surface waters and groundwaters are carbamazepine (CBZ), an antiepileptic and mood stabilizer, and sulfamethoxazole (SMX), a sulfonamide antibiotic commonly used in human and veterinary medicine [3]. Because a large fraction of these drugs are excreted unchanged in urine, they frequently persist in wastewater and natural waters [4]. Their presence, even at trace levels (typically in the ng·L^−1^ to low µg·L^−1^ range), has been repeatedly documented in global water systems [5]. Ecologically, these compounds can disrupt physiological and behavioral processes in aquatic organisms, affecting reproduction, development, and potentially promoting antibiotic resistance.

In South Africa, for example, CBZ and SMX are among a priority list of pharmaceuticals detected in surface and groundwater, yet regulatory limits for their discharge are still limited or nonuniform [2]. CBZ is persistent and poorly removed by conventional treatment systems, raising concerns over its continuous circulation in the environment [6]. Traditional analytical techniques for detecting CBZ and SMX such as gas chromatography (GC), planar chromatography [7], electrokinetic chromatography [8], LC‐MS tandem, and high‐performance liquid chromatography (HPLC) [9]. In contrast, electrochemical sensing offers a compelling alternative: low cost, portability, rapid response, and high sensitivity [10]. Nanomaterials and in particular manganese dioxide (MnO_2_) has shown considerable promise in improving the performance of electrochemical sensors. MnO_2_ is abundant, low in toxicity, redox‐active, and has tunable morphology (e.g., nanowires, nanoparticles), making it ideal for enhancing electrocatalytic activity and increasing electrode surface area [11].

Their nanostructured forms (nanoparticles, nanowires, mesoporous cubes, etc.) enable large surface‐area‐to‐volume ratios, facilitating rapid adsorption–desorption of target gases and yielding high sensitivity and low detection limits [12]. Seifi et al. [13] addressed this by optimizing the electrodeposition and post‐treatment of MnO_2_ films, substantially lowering the interfacial resistance and improving sensor performance. Gowda et al. [14] demonstrated the electrosensing of olanzapine using MnO_2_NP modified glassy carbon electrodes with a detection limit in the low nanomolar range. In a related study, the same authors developed a MnO_2_NP based chemical sensor for the antiviral drug valaciclovir, showing high sensitivity and good linearity [15]. More broadly, manganese oxide materials have been reviewed for their application in sulfonamide sensors, highlighting their tunable morphology, catalytic activity, and charge‐transfer properties [5]. However, a key limitation in many Mn‐oxide‐based sensors remains the relatively high charge‐transfer resistance associated with certain morphologies. In this study, we developed an electrochemical sensor based on manganese oxide nanoparticles modified screen‐printed carbon electrode (MnO_2_NPs/SPCE) for the detection of CBZ and SMX. Owing to the abundance and low cost of manganese, the proposed platform offers an affordable yet highly sensitive and selective sensing approach. The sensor's practical applicability was further demonstrated through successful detection of CBZ and SMX in real wastewater samples. Overall, the MnO_2_NPs/SPCE sensor provides an efficient and low cost solution for the simultaneous monitoring of these pharmaceutical pollutants.

## 2 Experimental

### 2.1 Reagents and Materials

All reagents and chemicals used in this study were of analytical grade (≥99% purity) and were used without any further purification. Potassium permanganate (KMnO_4_, ≥99%), manganese(II) sulfate monohydrate (MnSO_4_ · H_2_O, ≥99%), sodium hydroxide (NaOH, ≥98%), ethanol (C_2_H_5_OH, ≥99.5%), hydrochloric acid (HCl, 37%, AR grade), methanol (CH_3_OH, ≥99.8%), (CBZ, ≥99%), and (SMX, ≥99%) were all purchased from Sigma–Aldrich (South Africa). A 0.1 M phosphate buffer solution (PBS) at pH 7.0 was used as the supporting electrolyte. The PBS was prepared using the following components at their respective concentrations:

• Na_2_HPO_4_ (0.067 M)
• NaH_2_PO_4_ (0.033 M)

These concentrations were combined to achieve a total buffer strength of 0.1 M. The final pH of the PBS was adjusted to 7.0 using 0.1 M NaOH or 0.1 M HCl, as required. A 20 mM stock solution of CBZ and SMX was prepared in methanol and stored at 4°C until use.

### 2.2 Instrumentation and Measurement

The electrochemical measurements were performed on Emstat Blue 400 potentiostat (South Africa). Metrohm 110 Drop Sens SPCE were utilized as the working, reference, and counter electrodes. The electrochemical characterization of the bare and modified SPCE was performed by the cyclic voltammetry (CV) and electrochemical impedance spectroscopy (EIS) (Metrohm Autolab Potentiostat, South Africa). CV provides information about the redox properties and behavior of molecules or species in solution, while EIS is an analytical tool to study the interfacial behavior occurring on the surface of an electrode. The EIS experiment was performed in 0.1 M KCl solution containing 5 mM [Fe (CN)_6_]^3^^−/^^4^^−^ supporting electrolyte using the frequency range of 0.1 Hz–100 kHz and amplitude: 5–10 mV on an open circuit potential (OCP). The differential pulse voltammetry (DPV) method analyzing electrocatalytic activity toward different concentrations of CBZ and SMX. This technique was used to detect and quantify the electroactive species in solution. Then, the electrochemical performances toward the simultaneous determination of the two different pharmaceutical compounds were also investigated.

### 2.3 Fabrication of the MnO_2_NPs/SPCE Electrode

**FIGURE 1:**
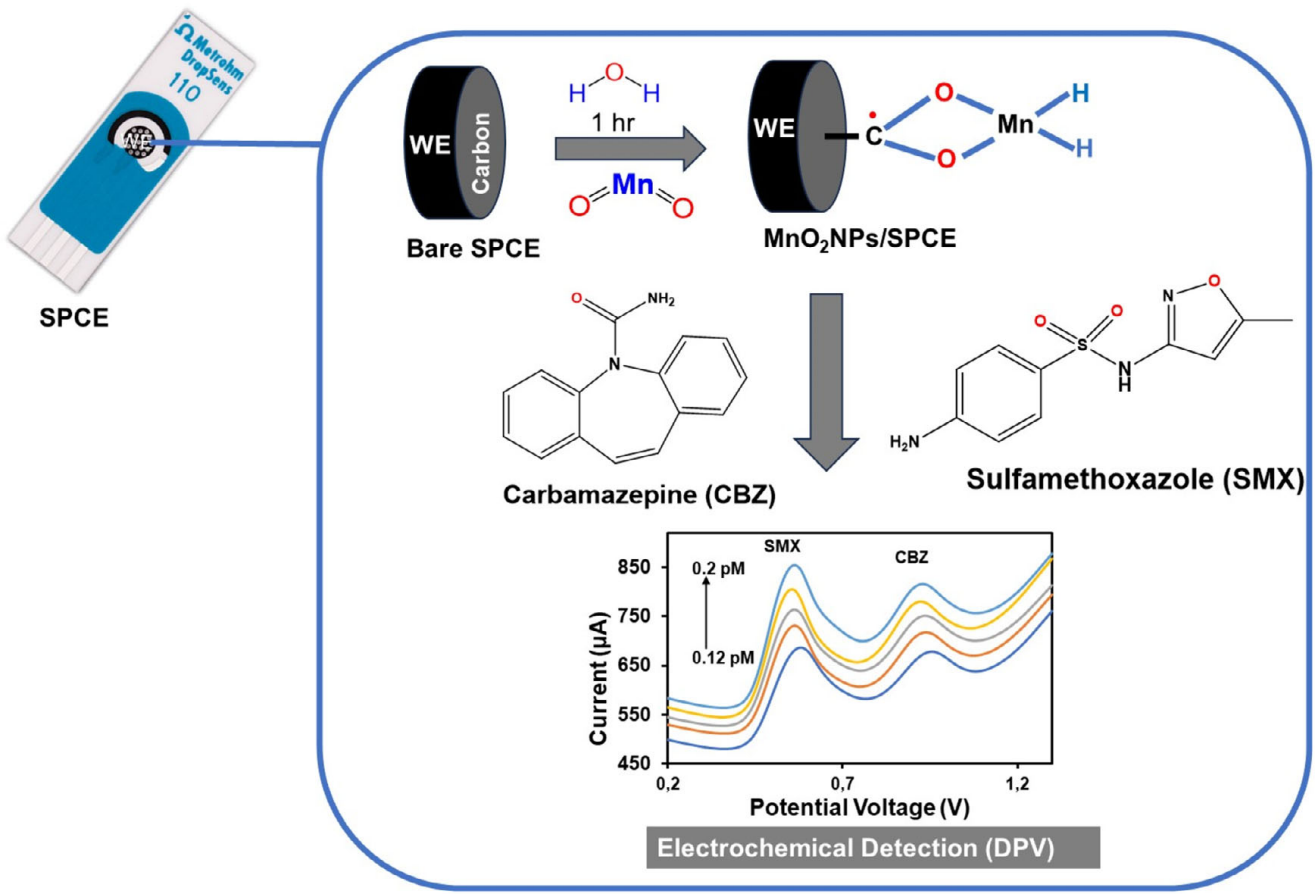
Schematic visualization of manganese oxide‐based electrochemical sensor for the detection of CBZ and SMX.

Prior to modification, the SPCE was thoroughly washed with double distilled water to remove any binding impurities. Then, about 5.0 mg of MnO_2_ powder were dispersed in 1 mL of double distilled water and then ultrasonicated for 15 min to obtain a homogeneous dispersion. Thus, 8.0 µL of MnO_2_ suspension was drop‐cast onto the surface of SPCE and allowed to dry at room temperature for 1h [16]. The successfully fabricated MnO_2_/SPCE was used for further electrochemical measurements in 0.1 M PBS (pH = 7.0) for CBX and SMX oxidation. The schematic representation of the modification process is shown in Figure 1.

### 2.4 Application of the Developed Sensor

The experimental procedure involved the following steps. Different concentrations of CBZ solutions were prepared in PBS, covering a range of concentrations to establish a calibration curve. The SPCE sensor was modified with MnO_2_NPs prior to experimentation to enhance its electrochemical performance and sensitivity toward CBZ detection. Each prepared CBZ solution was drop‐cast onto the surface of the modified electrode. DPV was employed to measure the electrochemical response of the sensor to each concentration of CBZ. The process of drop‐casting CBZ solutions onto the modified electrode and recording DPV responses was repeated for more than six trials to ensure the reliability and reproducibility of the results. The recorded DPV responses were analyzed to develop a calibration curve relating the sensor's response (current or peak height) to the concentration of CBZ in solution.

This calibration curve allows for the quantitative determination of CBZ concentrations in unknown samples. The limit of detection (LOD) of the MnO_2_NPs/SPCE sensor for CBZ and SMX was determined following Equation (1)

(1) $$\mathrm{LOD}=\frac{3\delta }{S}$$

where δ is the standard deviation of the blank (measured from multiple replicates of the blank response) and *S* is the slope of the calibration curve (obtained from the linear regression equation of the concentration vs. current response). The electrochemical response of the blank solution (without the analyte) was recorded at least ten times to determine the standard deviation (*σ*). The blank solution consisted of the supporting electrolyte PBS. Electrochemical measurements, e.g., DPV was performed for varying concentrations of CBZ and SMX. A plot of current (µA) versus concentration (µM) was generated. The linear portion of the calibration curve was used to determine the slope (*S*). The standard deviation (*σ*) from blank measurements was multiplied by 3 and divided by the slope *S* to obtain the LOD value.

### 2.5 Preparation of the Real Water Samples

Wastewater samples were collected from the Northern wastewater treatment plant (WWTP) situated in the Diepsloot suburb close to the N14, Gauteng Province, South Africa. It handles 400 ML of residential wastewater per day (ML/d) from the areas north of Hillbrow Ridge, which includes Alexandra, Randburg, Sandton, and some parts of Midrand and Roodeplaat. The samples were stored in the cooler box with ice, then transferred to the laboratory to be stored at 4°C before analysis. The wastewater samples were filtered with 0.45 µM. The water sample was filtered and fortified with the analytes of interest. Then, aliquots of 100 μL of each sample were diluted in 1000 μL supporting electrolyte solution, and the final solutions were analyzed [17].

## 3 Results and Discussion

### 3.1 Characterization for MnO_2_ Nanoparticles

The physicochemical and structural properties of the MnO_2_ nanoparticles used in this study were extensively characterized and previously reported by Mokaba et al. (2024) [11]. In that work, the MnO_2_ nanomaterial was synthesized and characterized using techniques including scanning electron microscopy (SEM), transmission electron microscopy (TEM), X‐ray diffraction (XRD), and Fourier transform infrared spectroscopy (FTIR). The characterization confirmed the successful formation of crystalline MnO_2_ nanoparticles with high‐surface area and a uniform morphology favorable for electrochemical applications. Given that the material synthesis and characterization were thoroughly detailed and published.

### 3.2 Electrochemical Analysis

#### 3.2.1 Influence of pH

**FIGURE 2:**
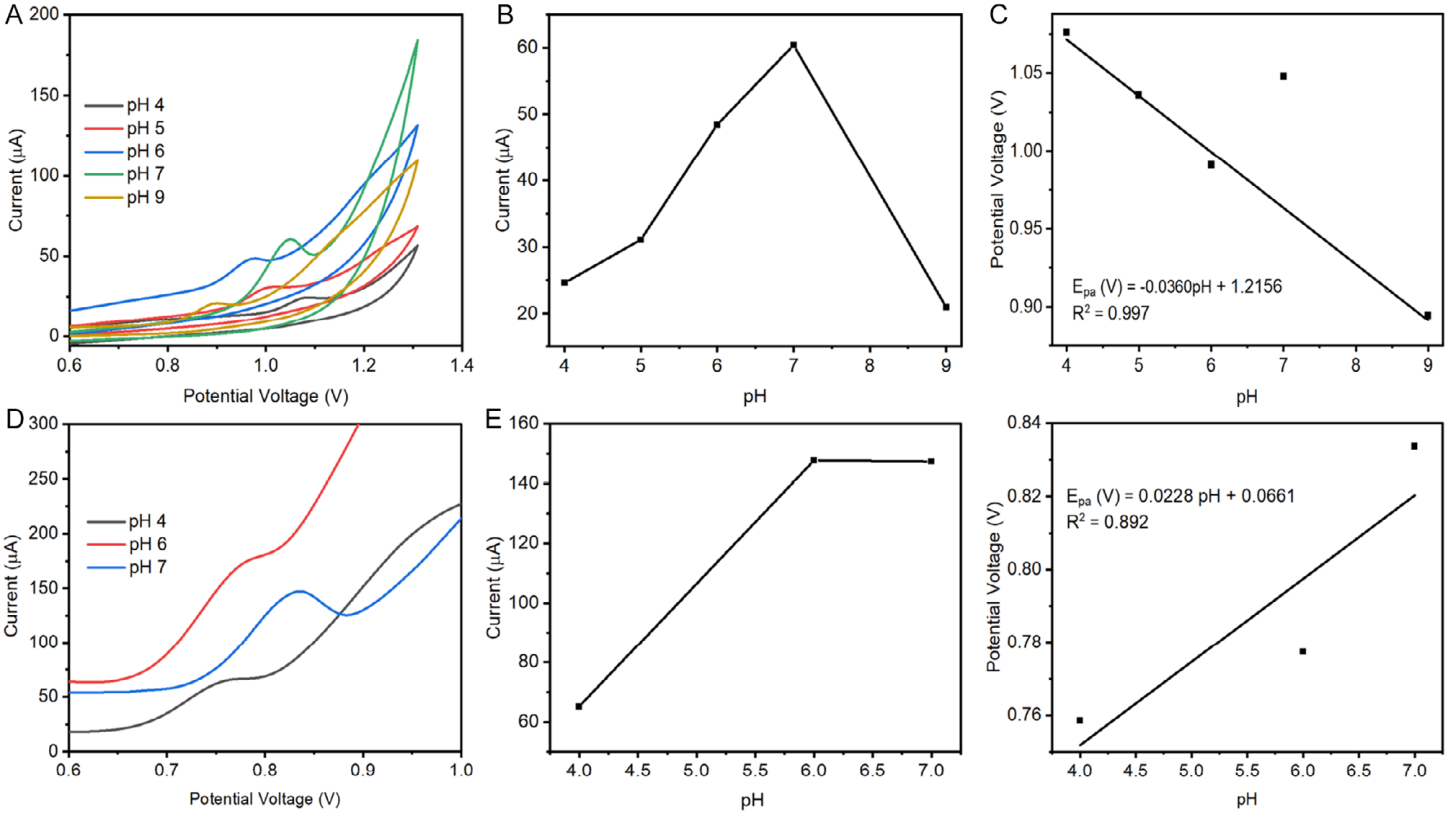
(A) CV response of the CBZ at MnO_2_NPs/SPCE at different pH range of 4–8 in the 0.1 M PBS, (B) a plot of pH versus current, (C) linear plot between pH versus peak potential, (D) DPV response of the SMX at MnO_2_NPs/SPCE at different pH range of 4,6–7, (E) a plot of pH versus current, and (F) linear plot between pH versus peak potential, all in the 0.1 M PBS.

**SCHEME 1:**
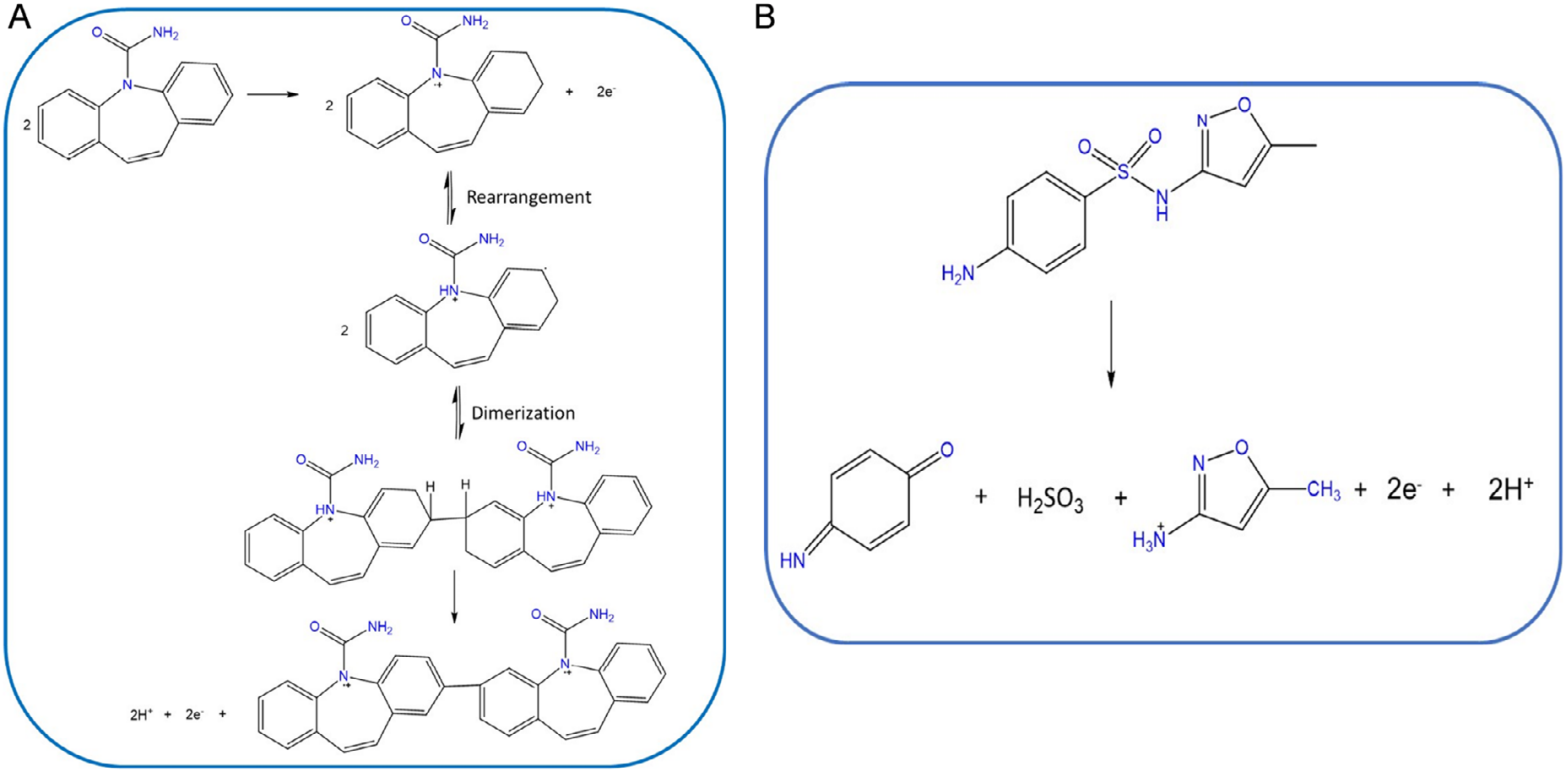
The proposed mechanism for (A) CBZ and (B) SMX oxidation on MnO_2_NPs/SPCE modified electrode.

The influence of pH on the electrochemical oxidation of CBZ provides important insight into the proton‐coupled electron transfer (PCET) mechanism occurring at the MnO_2_NPs/SPCE surface. As shown in Figure 2A,B CV was used to investigate the influence of pH (4.0–8.0) on the MnO_2_NPs/SPCE in 0.1 M PBS, the oxidation peak current of CBZ gradually increases from pH 4.0 to pH 7.0, where the maximum response is observed. This trend indicates that the electro‐oxidation of CBZ is highly sensitive to the protonation state of the molecule. At low pH values (4.0), CBZ exists predominantly in a protonated form, which may hinder its adsorption and electron transfer efficiency at the MnO_2_‐modified electrode surface due to increased solvation and weaker interaction with the electrode. As the pH increases, CBZ gradually shifts toward its neutral form, which is known to interact more favorably with hydrophobic or metal oxide surfaces, thereby enhancing electron transfer kinetics and increasing the anodic peak current. The slight negative shift in anodic peak potential (Epa) with increasing pH further confirms the involvement of protons in the oxidation process. This behavior is typical of PCET reactions, where the removal of protons becomes energetically easier as the solution pH increases, hence lowering the required oxidation potential. However, beyond pH 7.0, the decline in current suggests that further deprotonation of CBZ leads to a form less electrochemically active or less strongly adsorbed, reducing electron transfer efficiency [18]. Therefore, pH 7.0 was selected as optimal for subsequent experiments. A plot of the anodic peak potential (*E*_pa_) versus pH was determined with linear regression equation, *E*_pa_ = −0.0360 pH + 1.2156 and a correlation coefficient of *R* ^2^ = 0.997 (Figure 2C). The slope of *E*_pa_ versus pH can be used to estimate the electrochemical process within the electrode surface (Figure 2E,F). The experimentally observed slopes are closer to –0.030 V pH, which indicates that the electron–proton transfer processes for CBZ is not equal, implying a nonequimolar proton–electron involvement in the electrode reaction mechanism in Scheme 1A. This interpretation aligns with literature reports demonstrating that slopes near –0.030 V pH correspond to unequal numbers of protons and electrons transferred during the redox process [19].

On the other hand, DPV was used to investigate the influence of pH (4.0, 6.0, 7.0) on the electrochemical oxidation of SMX on MnO_2_NPs/SPCE electrode in 0.1 M PBS as shown in Figure 2D,E. The anodic peak current increased with an increase in pH and reached maximum current at pH 7.0. SMX is a sulfonamide compound with ionizable functional groups (amine and sulfonamide). Its electrochemical activity strongly depends on its protonation state [20]. At low pH (≈4), SMX is mostly protonated. Protonation can reduce adsorption and electron transfer at the electrode, resulting in lower current. SMX exists in its neutral or slightly deprotonated form, which interacts more effectively with the MnO_2_‐modified electrode and facilitates oxidation as we observed the maximum pH at 7. Only three pH values were evaluated (pH 4–7) because SMX's electrochemical response deteriorated significantly outside this window due to its acid–base speciation, which led to unstable or suppressed oxidation signals. However, to simultaneously determine CBZ and SMX, pH 7.0 was selected as the optimal for subsequence analysis. A plot of the anodic peak potential (*E*_pa_) versus pH was determined with linear regression equation, *E*_pa_ = −0.0228 pH + 0.0661 and a correlation coefficient of *R* ^2^ = 0.892 (Figure 2F). Scheme 1B shows that the electron–proton transfer processes for both SMX is not equal.

### 3.3 Effect of Scan Rate

**FIGURE 3:**
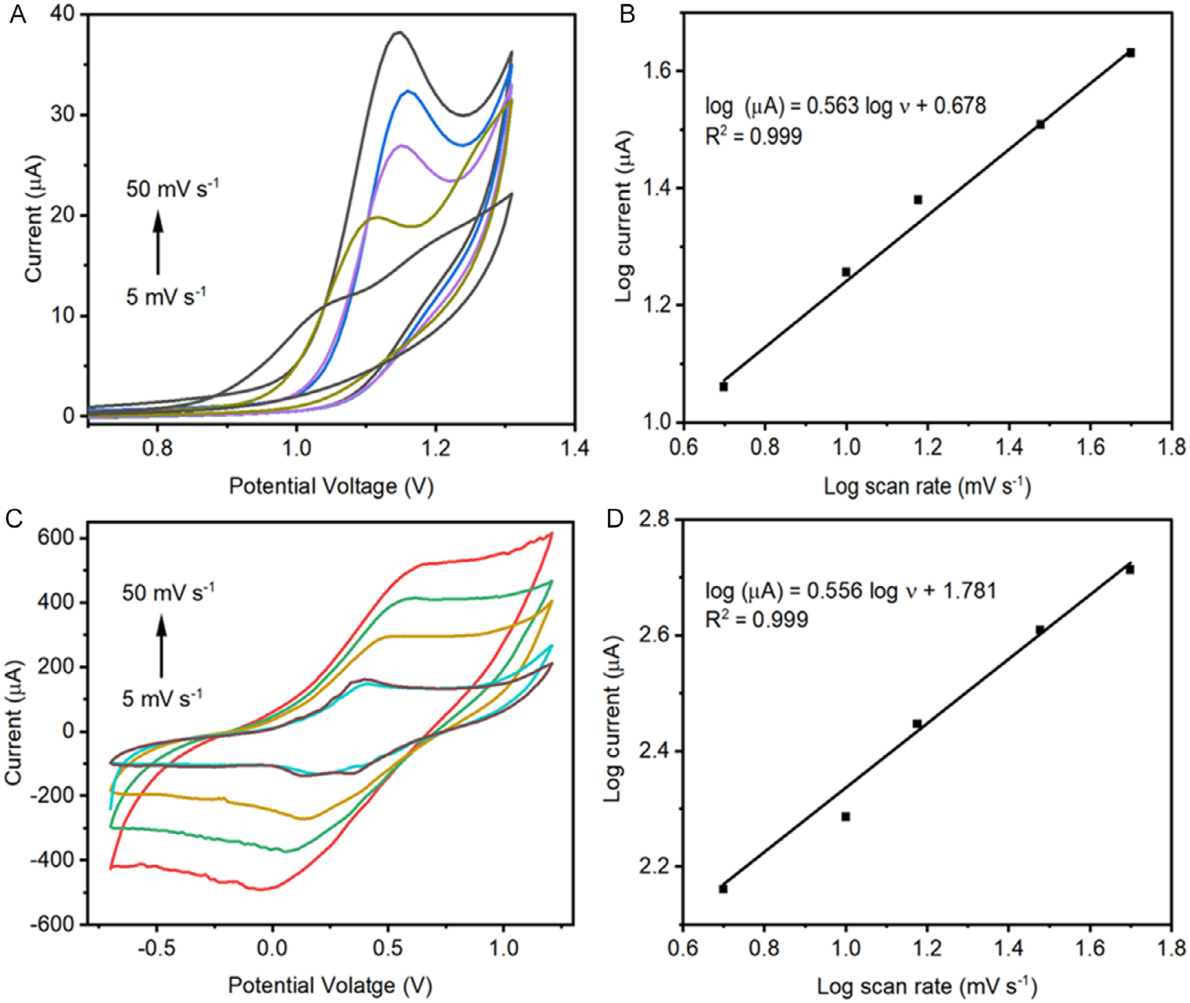
CV measurements of the MnO_2_NPs/SPCE at different scan rate range 5–50 mV/s and linear plot relationship (A,B) carbamazepine (C,D) sulfamethoxazole 0.1 M PBS, pH = 7.0.

The effect of scan rate on the oxidation of CBZ and SMX at MnO_2_NPs/SPCE modified electrode was examined by varying the scan rate from 5−50 mV s^−1^ using CV, respectively (0.1 M PBS, pH = 7.0). The anodic peak current (*i*_pa_) of CBZ increased with an increase in scan rate, along with a positive shift in peak potential, confirming that the oxidation of CBZ on MnO_2_NPs/SPCE is irreversible process [21]. (Figure 3A) The relationship between the log of scan rate and log of peak current yield a linear regression with equation log I (µA) = 0.563 log V + 0.678 and correlation coefficient *R* ^2^ = 0.999. This confirms that CBZ oxidation is primarily governed by diffusion process, where the transport of molecules from bulk solution to the electrode limits the current (Figure 3B) [22].

Similarly, the effect of scan rate upon the electrochemical oxidation of SMX was also investigated using the same condition as that of CBZ. As can be seen, the corresponding oxidation peak current increases relative to an increase in scan rate, along with a gradual potential shift toward a positive value. The plot between log of scan rate against log of peak current displays the linear relationship with regression line log I (µA) *=* 0.556 log + 1.781, *R* ^2^ = 0.999 (Figure 3D) However, a quasireversible electrochemical process for SMX detection was noticed. This could be attributed to the hindered electron transfer during the SMX oxidation process, which takes place to an extent that still allows its detection. Overall, these results demonstrate that while diffusion dominates the electrochemical response of both analytes. The MnO_2_NPs/SPCE effectively facilitates the oxidation of both compounds, providing clear and reliable peak currents for analytical applications. CV studies of SMX (Figure 3C) reveal that both the anodic (*E*_ap_) and cathodic (*E*_cp_) peak potentials shift in opposite directions as the scan rate increases from 5 to 50 mV s^−1^. The peak‐to‐peak separation (Δ*E*_p_ = *E*_ap_ – *E*_cp)_ also increases with scan rate, indicative of a quasireversible electron transfer process. For a fully reversible one‐electron redox couple, Δ*E*_p_ is expected to be ≈59 mV; however, the observed Δ*E*_p_ values for SMX range from 0.13 to 0.39 V and increase with scan rate, suggesting slower electron transfer kinetics and confirming quasireversible behavior. The electrode's active surface area is calculated from the Randles–Ševčík Equation (2)

(2) $$I_{p}=2.69\times 10^{5}(n^{3/2})(A)(C)(D^{1/2})(\vee^{1/2})$$

where *I*_p_ is the peak current, *n* is the number of electron transfers, *A* is the active surface of the electrode, *D* is the diffusion coefficient of the electroactive species in the electrolyte (3.64 × 10^−6^ cm^2^/s), *C* refers to the concentration of the redox probe, and ∨ is the scan rate [18].

The electroactive surface area of the electrodes was determined using the reversible redox couple K_4_ [Fe (CN)_6_]/K_3_ [Fe (CN)_6_] in 0.1 M PBS and applying the Randles–Ševčík equation

(3) $$I_{p}=2.69\times 10^{5}\times n^{\frac{3}{2}}\times A\times D^{\frac{1}{2}}\times C\times v^{\frac{1}{2}}$$

Rearranged to solve for *A*

(4) $$A=\frac{I_{p}}{2.69\times 10^{5}\times n^{\frac{3}{2}}\times D^{\frac{1}{2}}\times C\times v^{\frac{1}{2}}}$$

Using the following parameters:

*I*_p_ (Modified) = 38 µA = 38 × 10^−6^ A

*I*_p_ (bare) = 10.47 µA = 10.47 × 10^−6^ A

*n* = 1 (for the Fe (CN)_6_^3−^/Fe (CN)_6_^4−^ system)

*D* = 3.64 × 10^−6^ cm^2^/s

*C* = 18.7 mM (concentration of redox probe) = 1.87  × 10^−5^ mol/cm^3^

*ν* = 50 mV/s = 0.05 V/s

The calculated active surface areas are:

• Modified electrode: 0.01771 cm^2^
• Bare electrode: 0.004879 cm^2^

These results indicate that modifying the SPCE with MnO_2_ nanoparticles significantly enhances the electroactive surface area, which contributes to the improved electrochemical performance of the sensor.

### 3.4 Electrochemical Behavior of CBZ and SMX on MnO_2_NPs/SPCE

**FIGURE 4:**
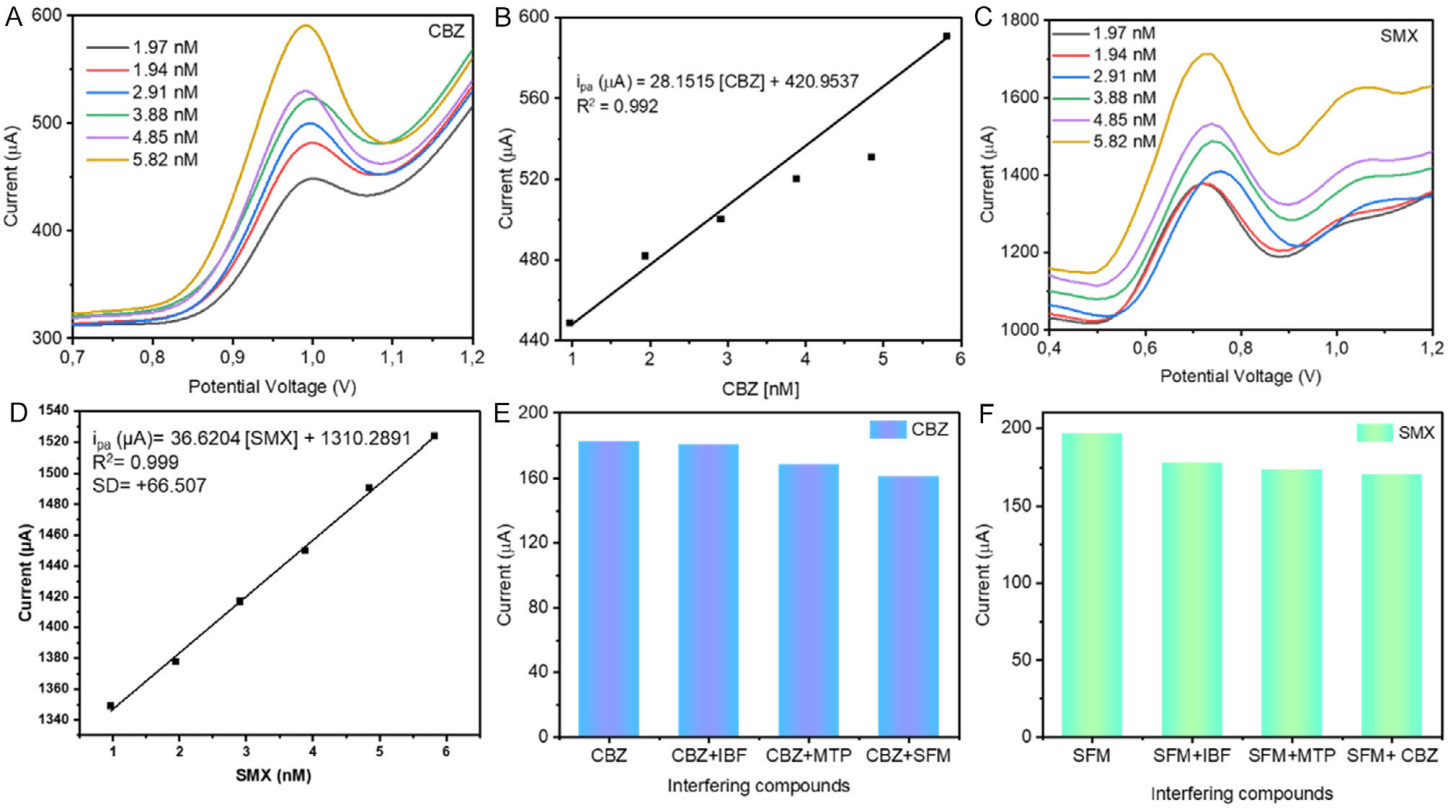
DPV signals of MnO_2_NPs/SPCE (A) on the determination of CBZ. (B) The calibration plot between concentrations versus anodic peak currents of CBZ, (C) on the determination of SMX, (D) the calibration plot between concentrations versus anodic peak currents of SMX, from 0.97 to 5.82 nM in 0.1 M PBS solution (pH 7.0), (E) peak current responses of 20 nM CBZ in the presence of 20 nM of SMX, IBU, and MP, and (F) 20 nM SMX in the presence of 20 nM of CBZ, IBU and MTP.

The DPV was used to examine the electrochemical behavior of CBZ and SMX on MnO_2_NPs/SPCE under the optimum conditions (0.1 M PBS, pH = 7.0), while varying concentrations from 0.97 to 5.82 nM (Figure 4). The oxidation peak current of CBZ increases with an increase in its concentration from 0.97 to 5.82 nM (Figure 4A). A plot between anodic peak current and concentration of CBZ revealed a linear relationship with regression equation *i*_pa_ (μA) = 33.243 [CBZ] + 409.12 and correlation coefficients of *R* ^2^ = 0.9635, (Figure 4B). Similarly, Figure 4C shows the DPV response of SMX on MnO_2_NPs/SPCE in 0.1 M PBS solution. Upon the increase in SMX concentration, a progressive increase in peak current was observed. The calibration plot of SMX concentration against peak current display a linear regression equation plot were: *i*_p_ (μA) = 34.465 [SMX] + 1309.2 and correlation coefficient *R* ^2^ = 0.9885 as shown in Figure 4D. The peak potential shift observed in the DPV voltammograms (Figure 4A for CBZ and Figure 4C for SMX) is due to the different oxidation mechanisms, functional groups, and electron transfer kinetics of the two compounds. undergoes electro‐oxidation primarily through the oxidation of the amide or heterocyclic functionalities. The CBZ undergoes electro‐oxidation primarily through the oxidation of the amide or heterocyclic functionalities while SMX oxidizes through the sulfonamide and aniline moieties because these molecular groups oxidize at different energy levels, the peak potentials naturally differ. These differences reflect the intrinsic electron transfer kinetics and energy levels of the redox‐active sites in each molecule. The MnO_2_NPs facilitate these oxidation reactions by providing a highly conductive and catalytically active surface, enhancing electron transfer and sensitivity. The MnO_2_NPs/SPCE sensor demonstrates a high sensitivity, as evidenced by the pronounced peak current even at low nanomolar concentrations. Good linearity, ensuring accurate quantification over the tested concentration range. Distinct and well‐resolved peak potentials, allowing simultaneous detection of CBZ and SMX without interference. Superior performance compared to previously reported sensors (Table 1), offering lower detection limits and comparable or wider linear ranges. The results highlight that the MnO_2_NPs/SPCE electrode effectively facilitates diffusion‐controlled, proton‐coupled electron transfer reactions for both CBZ and SMX. The sensor's performance makes it highly suitable for environmental monitoring of pharmaceutical pollutants, where low‐level detection and selectivity are critical.

**TABLE 1:** Comparison of the electrochemical performance of MnO_2_NPs/SPCE sensor to previously reported sensor for the determination of CBZ and SMX.

Analytes of interest	Electrode used	Linear range, nM	LOD, nM	Reference
CBZ	MWCNT/GCE	1.00–16.00	4.00 × 10^4^	[22]
	Au/graphene‐AuNPs/GCE	5.00–100	3.00 × 10^6^	[23]
	Fullerene‐C60/GCE	5.00–10.00	7.60 × 10^4^	[24]
	Ag/TiO_2_/CPE	2.50–100	8.60 × 10^5^	[25]
	Ce‐ZnO/rGO/GCE	0.10–100	10.00 × 10^3^	[26]
	MnO_2_NPs/SPCE	0.97–5.82	0.106	This work
SMX	MWCNT‐SbNP	1.00–7.00	2.00 × 10^4^	[27]
	SHL‐GP/WP	5.00–100	4.00 × 10^5^	[28]
	MWCNT/PBnc/SPE	1.00–10.00	4.00 × 10^4^	[29]
	AArGO‐modified electrode	5.00–50.00	4.00 × 10^4^	[30]
	MnO_2_NPs/SPCE	0.97–5.82	0.082	This work

Abbreviations: MWCNT, multiwalled carbon nanotube; GCE, glassy carbon electrode; Au‐ gold, AuNPs‐gold nanoparticles; Ag, silver; TiO_2_, titanium dioxide; CPE, carbon paste electrode; Au@AgPdNPs, Au core AgPd shell nanoparticles; *β*‐CD‐IL, macrocyclic *β*‐cyclodextrin‐ionic liquid; Ce, Cerium; ZnO, Zinc Oxide; rGO, reduced graphene oxide. MWCNT‐ SbNP, carbon nanotubes modified with antimony nanoparticles in a paraffin composite electrode; Paper‐based rGNR, paper‐based fully printed electrochemical sensor with reduced graphene nanoribbons; SHL‐GP/WP, shellac‐based graphite on waterproof paper; MWCNT/PBnc/SPE, multiwalled nanotubes decorated with Prussian blue nanotubes modified screen‐printed electrode**;** AArGO‐modified electrode, ascorbic acid reduced graphene oxide‐ modified electrode. These values were converted to nM.

### 3.5 Interference Studies

Selectivity is a crucial parameter in evaluating the applicability of an electrochemical sensor, as it reflects the sensor's ability to distinguish the target analyte from coexisting substances in complex matrices. In this study, the MnO_2_NPs/SPCE sensor was tested for its ability to selectively detect CBZ and SMX in the presence of potentially interfering compounds such as ibuprofen, metoprolol, and other structurally related pharmaceuticals at 20 nM (Figure 4E,F). The DPV results show that the oxidation peak currents of both CBZ and SMX were largely unaffected by the presence of these interfering species, indicating minimal cross‐reactivity. This high selectivity can be attributed to several electrochemical factors. Each analyte possesses unique redox‐active functional groups that oxidize at specific potentials. CBZ undergoes oxidation primarily through its amide or heterocyclic moieties, while SMX oxidizes via sulfonamide and aniline groups. The interfering compounds have different functional groups with oxidation potentials that do not overlap with CBZ or SMX, resulting in no significant interference signals. The MnO_2_ nanoparticles provide a high‐surface‐area, catalytically active interface that enhances electron transfer for the target analytes. Their surface interactions are selective toward molecules with specific adsorption affinities and redox characteristics, further reducing interference from other compounds. The well‐defined and separate anodic peaks for CBZ and SMX in DPV allow for precise differentiation of signals, even in the presence of structurally similar molecules. The excellent anti‐interference ability highlights that the MnO_2_NPs/SPCE sensor not only facilitates diffusion‐controlled, PCET for the target analytes but also discriminates effectively based on molecular structure and redox potential, making it highly suitable for simultaneous and selective detection of CBZ and SMX in complex environmental samples [31]. The relative standard deviation (RSD%) is a statistical measure of the precision and reproducibility of analytical measurements. In your study, the RSD values were 5.82% for CBZ and 6.47% for SMX in the presence of potential interfering species. These relatively low RSD values indicate that the MnO_2_NPs/SPCE sensor produces consistent and repeatable peak currents even when other compounds are present.

### 3.6 Stability Studies

**FIGURE 5:**
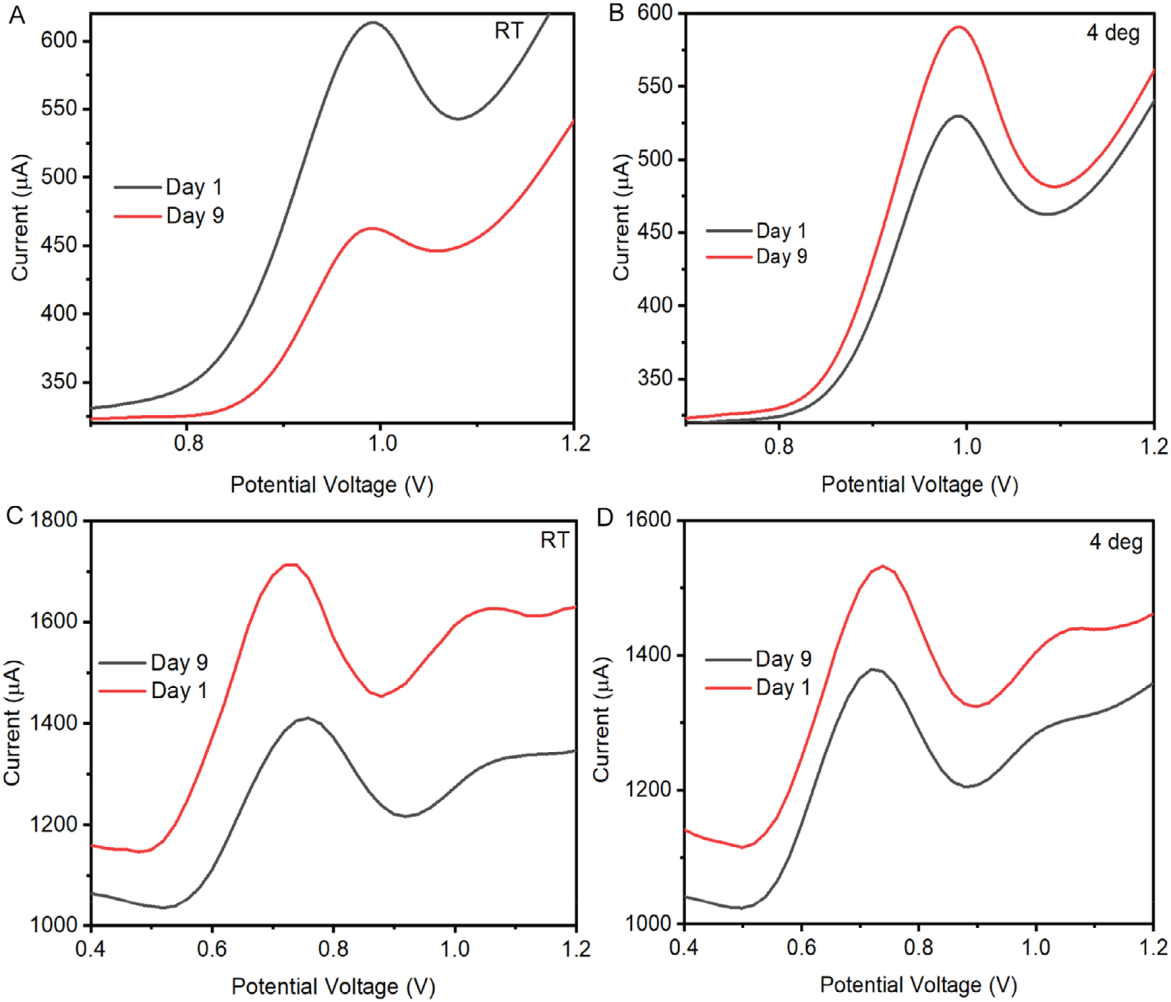
Differential pulse voltammetry (DPV) responses of MnO_2_NPs/SPCE to CBZ and SMX on day 1 and day 9 after storage, respectively (A,C) at room temperature (B,D) at 4°C.

The long‐term stability of the MnO_2_NPs/SPCE sensor is crucial for practical applications, especially for environmental monitoring where sensors may be stored or reused over extended periods. Stability was evaluated over 9 days under two storage conditions: room temperature and refrigeration (4°C) using DPV measurements of CBZ and SMX, as shown in Figure 5A,C. When stored at room temperature, the MnO_2_NPs/SPCE retained only 67% of its initial current response for both CBZ and SMX. This decrease reflects the agglomeration of MnO_2_ nanoparticles, which is enhanced by higher temperatures. Loss of surface area due to nanoparticle clustering. Reduced availability of electroactive sites for analyte adsorption and weakened electron transfer pathways as the MnO_2_ network becomes less conductive. From an electrochemical standpoint, the diminished current indicates a slower charge‐transfer rate, consistent with partial blocking of catalytic sites and increased charge‐transfer resistance. As the electrode surface becomes less accessible, oxidation of CBZ and SMX becomes less efficient, resulting in lower peak currents. Refrigerate storage allowed the sensor to retain 84% of its initial response after 9 days, significantly higher than room‐temperature storage, as shown in Figure 5B,D. This improved performance can be attributed to reduced thermal energy, which slows nanoparticle movement and prevents excessive agglomeration. Preserve porosity and surface morphology of the MnO_2_NPs coating. Stable redox‐active Mn^4+^/Mn^3+^ sites, which remain available for electron transfer. Slower degradation of the SPCE binder and MnO_2_ film. Electrochemically, maintaining a high proportion of electroactive surface area ensures efficient charge‐transfer kinetics, lower charge‐transfer resistance and stable oxidation signals for CBZ and SMX. Thus, refrigeration slows structural and chemical changes, promoting consistent electrochemical behavior [32]. The overall stability is sufficient for real‐world environmental sensing applications, highlighting the robustness and practical viability of the MnO_2_NPs/SPCE system [33]. Therefore, MnO_2_NPs/SPCE sensor demonstrates good long‐term stability, particularly under refrigerated storage, retaining 84% of its initial electrochemical performance.

### 3.7 Simultaneously Detection of CBZ and SMX Using MnO_2_NPs/SPCE Sensor

**FIGURE 6:**
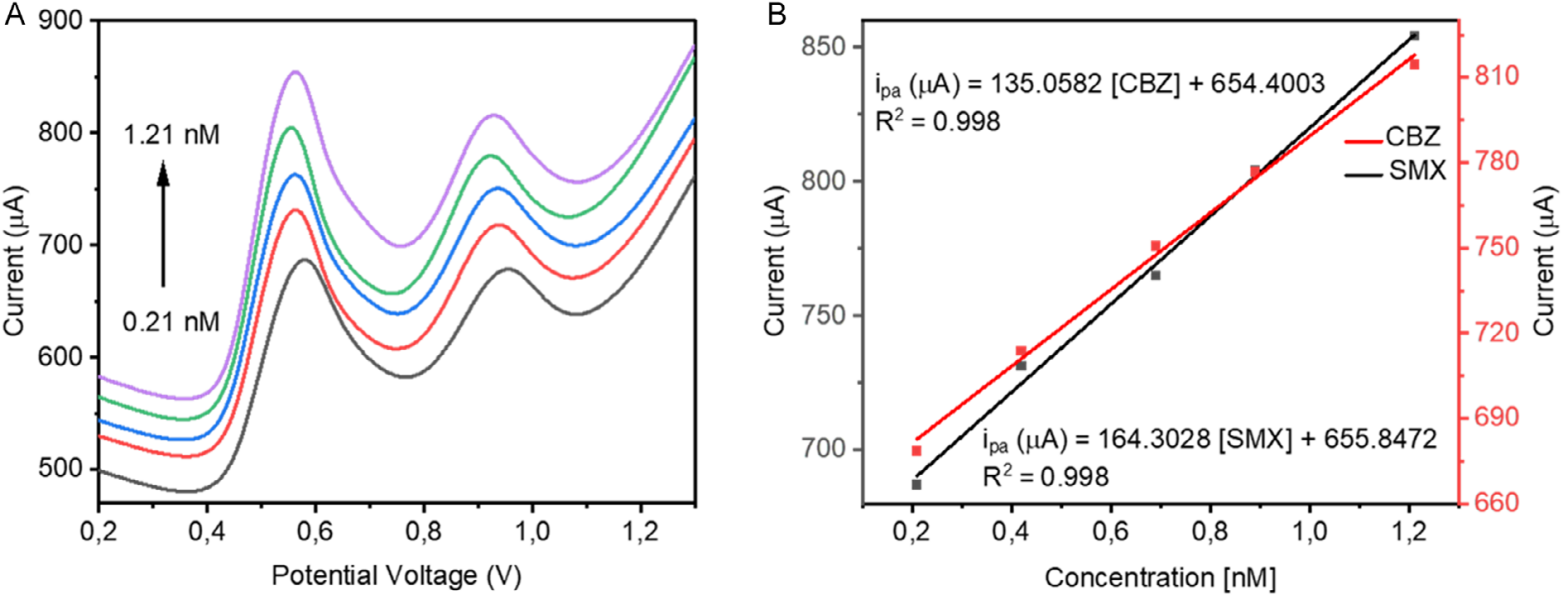
DPV voltammograms for the MnO_2_NPs/SPCE under optimized parameters in 0.1 M PBS (pH 7) for the simultaneous detection of SMX and CBZ.

The MnO_2_NPs/SPCE sensor demonstrates excellent performance for the simultaneous detection of CBZ and SMX, as evidenced by proportional increases in peak currents with analyte concentrations and strong linear correlations (*R* ^2^ = 0.998 for both compounds). The slight negative shift in peak potentials during simultaneous detection suggests that interanalyte interactions and surface effects slightly modify the redox behavior compared to individual detection. This could arise from electron density redistribution at the electrode surface or transient surface adsorption phenomena, slightly lowering the energy required for the redox processes, as illustrated in Figure 6. MnO_2_ nanoparticles provide a high‐surface‐area and conductive platform, facilitating rapid electron transfer for both CBZ and SMX. The improved kinetics reduce overpotential and increase peak currents, contributing to higher sensitivity [32]. The observed reduction potentials (+0.59 V for SMX, +0.98 V for CBZ) indicate that electron transfer is occurring efficiently at the electrode surface, possibly involving PCET or the temporary formation of adsorbed intermediate species. The MnO_2_NPs may mediate these redox reactions by providing catalytic active sites that stabilize transition states or reactive intermediates [34]. The linear increase of peak currents with concentration and high correlation coefficients indicate that diffusion governs the transport of analytes to the electrode surface, while adsorption interactions with MnO_2_ enhance local analyte concentration, improving sensitivity. The slightly higher sensitivity observed for SMX (164.3 nM) compared to CBZ (135.1 nM) suggests stronger adsorption or more favorable orientation of SMX molecules at the MnO_2_NPs surface, as shown in Figure 6B. This may be due to structural factors, such as sulfonamide and aniline groups in SMX interacting more effectively with oxygen‐containing functional groups on MnO_2_. The MnO_2_NPs/SPCE sensor exhibits fast electron transfer kinetics, strong analyte–surface interactions, and catalytic activity, which collectively result in high sensitivity, excellent linearity, and low detection limits. The combination of diffusion‐controlled transport and surface‐mediated redox processes underlies the superior performance, making the sensor highly suitable for trace‐level environmental or pharmaceutical monitoring of CBZ and SMX. The *R* ^2^ value of 0.998 for both analytes confirm a strong linear correlation, indicating high accuracy and reliability.

### 3.8 Real Water Samples Analysis

The applicability of the MnO_2_NPs/SPCE sensor for practical water analysis was assessed using effluent samples collected from a WWTP. After filtration to remove suspended solids and minimize matrix effects, known concentrations of CBZ and SMX were spiked into the samples and analyzed using DPV. The DPV responses showed a proportional increase in peak currents with the increasing concentrations of each analyte, demonstrating that the sensor maintained its sensitivity even in a complex matrix. The percentage recovery values ranged between 95% and 110% for CBZ and 90% and 105% for SMX, as shown in Table 2, indicating that the sensor accurately quantified both compounds within acceptable analytical limits. Recoveries within the 90%–110% range are considered good for trace‐level detection, especially in environmental matrices where organic matter, ions, and other pharmaceutical residues can interfere with electron transfer processes. The RSDs below 5% further confirm the high precision and repeatability of the measurements. Wastewater contains competing organic molecules and ions that may adsorb onto the electrode surface. However, the MnO_2_NPs/SPCE maintained high recovery rates, indicating that MnO_2_ nanoparticles provide selective interaction sites for CBZ and SMX and competing compounds did not significantly disrupt the redox processes of the target analytes. This aligns with earlier interference studies showing minimal peak distortion in the presence of coexisting pharmaceuticals. MnO_2_ nanoparticles enhance electron transfer kinetics, ensuring efficient redox reactions even in complex media. Signal amplification, leading to clearly resolved peaks despite matrix components. This catalytic property allows CBZ and SMX to retain strong electrochemical signatures in wastewater samples. The linear DPV response despite the matrix indicates that analytes diffused effectively to the electrode surface. The MnO_2_‐modified surface was not blocked by wastewater constituents. This stability is crucial for environmental monitoring applications. The high recovery rates, strong linearity, and low RSD values confirm that the MnO_2_NPs/SPCE sensor performs reliably in real wastewater samples. These results demonstrate its potential for on‐site, simultaneous detection of CBZ and SMX at trace levels, even in complex environmental matrices. The superior performance is attributed to the electrocatalytic properties of MnO_2_NPs, strong anti‐interference ability, and excellent stability of the SPCE platform.

**TABLE 2:** Electrochemical detection of CBZ and SMX in real water samples using MnO_2_NPs/SPCE.

Analyte of Interest	Samples	Added, pM	Found, pM	% Recovery	RSD%
CBZ	1	0.21 ± 0.10	0.20 ± 0.13	95	3.45
	2	0.29 ± 0.10	0.31 ± 0.13	107	4.71
	3	0.42 ± 0.10	0.45 ± 0.13	110	4.88
SMX	1	0.21 ± 0.15	0.20 ± 0.12	90	5.60
	2	0.29 ± 0.15	0.30 ± 0.12	103	2.40
	3	0.42 ± 0.15	0.43 ± 0.12	105	1.66

*Note:* SD standard deviation of three replicate determinations (*n* = 3).

## 4 Conclusion

In this study, a novel MnO_2_NPs‐modified SPCE (MnO_2_NPs/SPCE) was successfully developed for the highly sensitive and selective electrochemical detection of CBZ and SMX. The innovation of this work lies in the use of MnO_2_ nanoparticles to enhance electron transfer, increase the active surface area, and improve catalytic activity toward the oxidation of both pharmaceuticals, enabling simultaneous detection with high precision. The MnO_2_NPs/SPCE sensor demonstrated excellent analytical performance, achieving low detection limits of 0.106 nM for CBZ and 0.082 nM for SMX, along with wide linear ranges and strong signal stability. Compared with previously reported electrochemical sensors for pharmaceutical contaminants, the developed system shows superior sensitivity and lower detection limits, highlighting the effectiveness of MnO_2_NPs in enhancing electrochemical activity. Real wastewater analysis further confirmed the sensor's practical applicability, with satisfactory recovery values ranging from 95% to 110% for CBZ and 90% to 105% for SMX. These results are consistent with, and in some cases superior to, earlier studies, demonstrating that the modified electrode performs reliably even in complex matrices. Overall, the MnO_2_NPs/SPCE sensor represents a promising platform for on‐site, real‐time monitoring of pharmaceutical pollutants in aquatic environments. Its excellent sensitivity, stability, and simplicity suggest strong potential for broader environmental surveillance, water quality monitoring, and future integration into portable sensing devices for routine contaminant detection.

## 5 Future Work

Expanding the investigation to include additional pharmaceutical compounds with similar functional groups (e.g., other sulfonamides and anticonvulsants) and common inorganic ions (e.g., Na^+^, K^+^, Ca^2+^, Cl^−^, NO_3_^−^). This will provide further insights into the selectivity and robustness of the sensor, ensuring its applicability in complex real‐world matrices.

## Author Contributions

P.L.M. and C.N. wrote initial main manuscript, P.N.M., A.L., M.W.H., and U.F. revised the manuscript. All authors reviewed.

## Funding

This research was supported by the Institute for Nanotechnology and Water Sustainability, National Research Foundation of South Africa (138077), Department of Education (1766−22).

## Conflicts of Interest

The authors declare no conflicts of interest.

## Data Availability

The data that support the findings of this study are available from the corresponding author upon reasonable request. This includes raw data, and any supplementary information necessary for replicating the results. Data sharing is subject to ethical approvals and privacy restrictions where applicable.
